# Supplementary material for: Development and Validation of a Deep-Learning-Based Algorithm for Detecting and Classifying Metallic Implants in Abdominal and Spinal CT Topograms
Source: Diagnostics (Basel). 2024 Mar 22;14(7):668. doi: 10.3390/diagnostics14070668 (PMC11011303; doi:10.3390/diagnostics14070668)

Title: Development and validation of a deep learning-based algorithm for detecting and classifying metallic implants in abdominal and spinal CT topograms

Supplementary Figure S1. The neural network architecture

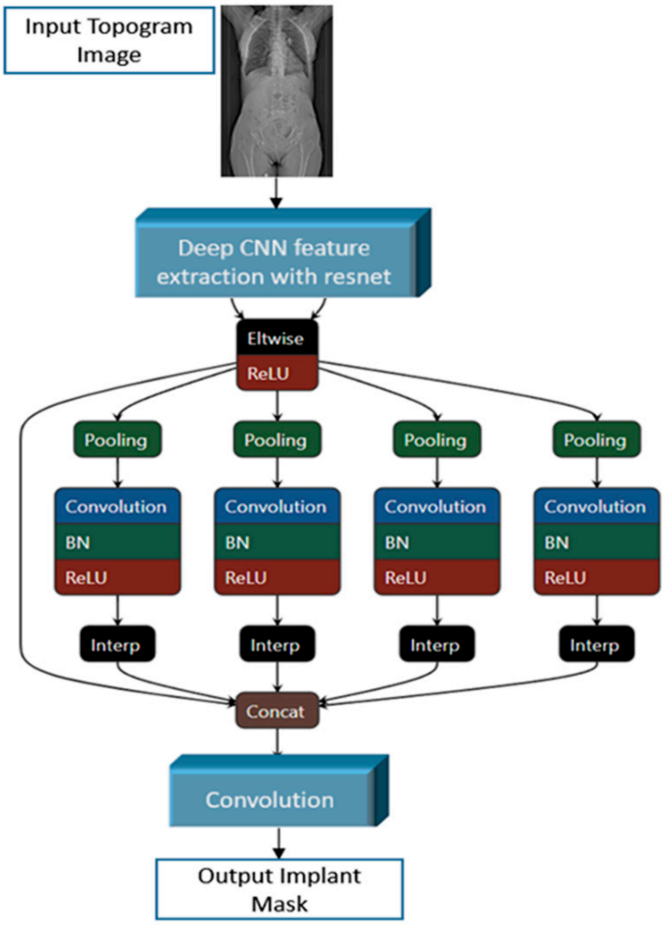

Supplementary Figure S2. Example annotation for a hip implant

The result of the detection algorithm shows the contour of implant and number of metal-detected pixel row indicated as a graph on the left side of the patient.

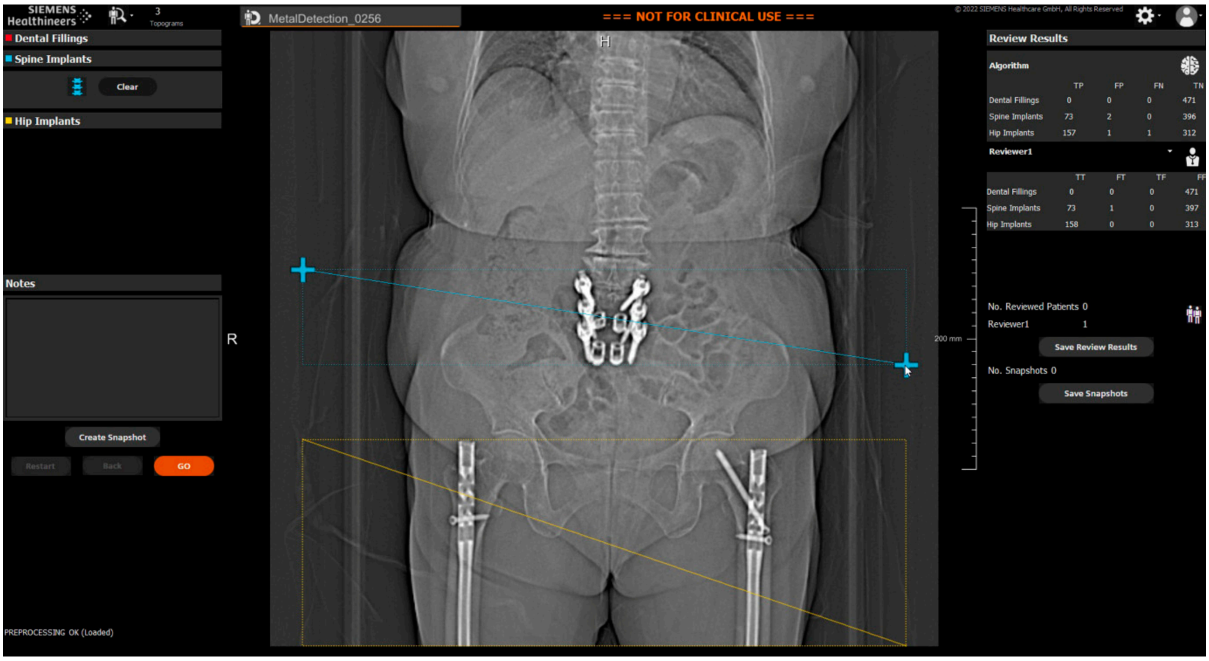

Supplementary material: statistical analysis

True positive (TP): Number of detected topogram pixel rows inside the annotated range

False positive (FP): Number of detected topogram pixel rows outside the annotated range

True negative (TN): Number of undetected topogram pixel rows outside the annotated range

False negative (FN): Number of undetected topogram pixel rows inside the annotated range

The per-patient performance metrics were calculated based on TP, FP, FN, and TN from pixel row counts as follows:

$$\boldsymbol{TP_{pat}} = \begin{cases} \mathbf{1}, \boldsymbol{if\ TP_{row} > FP_{row} + FN_{row}} \\ \mathbf{0}, \boldsymbol{otherwise} \end{cases}$$

$$\boldsymbol{FP_{pat}} = \begin{cases} \mathbf{1}, \boldsymbol{if\ TP_{row} \leq FP_{row} + FN_{row}\ and\ FP_{row} > FN_{row}} \\ \mathbf{0}, \boldsymbol{otherwise} \end{cases}$$

$$\boldsymbol{FN_{pat}} = \begin{cases} \mathbf{1}, \boldsymbol{if\ TP_{row} \leq FP_{row} + FN_{row}\ and\ FN_{row} > FP_{row}} \\ \mathbf{0}, \boldsymbol{otherwise} \end{cases}$$

$$\boldsymbol{TN_{pat}} = \begin{cases} \mathbf{1}, \boldsymbol{if\ TP_{row} + FP_{row} + FN_{row} = 0} \\ \mathbf{0}, \boldsymbol{otherwise} \end{cases}$$

The parameters of diagnostic performance were assessed per pixel row and per patient as follows:

Sensitivity = $\boldsymbol{TP}/(\boldsymbol{TP}+\boldsymbol{FP})$

Specificity = $\boldsymbol{TN}/(\boldsymbol{TN}+\boldsymbol{FP})$

Positive predictive value (PPV) = $\boldsymbol{TP}/(\boldsymbol{TP}+\boldsymbol{FP})$

Negative predictive value (NPV) = $\boldsymbol{TN}/(\boldsymbol{TN}+\boldsymbol{FN})$

Accuracy = $(\boldsymbol{TP}+\boldsymbol{TN})/(\boldsymbol{TP}+\boldsymbol{FP}+\boldsymbol{FN}+\boldsymbol{TN})$

Intersection over union (IoU) = $(\boldsymbol{TP})/(\boldsymbol{TP}+\boldsymbol{FP}+\boldsymbol{FN})$

Supplementary Table S1. External validation for abdomen CT anteroposterior topogram

	Spine implants				Hip implants			
	**DLAp-r**	**DLA**	**Reader 1**	**Reader 2**	**DLAp-r**	**DLA**	**Reader 1**	**Reader 2**
**Sensitivity (%)**	94.6	93.6 (58/62) [84.6-97.5]	93.6 (58/62) [84.6-97.5]	98.4 (61/62) [91.4-99.7]	78.1	97.3 (36/37) [86.2-99.5]	97.3 (36/37) [86.2-99.5]	100 (37/37) [90.6-100]
**Specificity (%)**	99.9	*† **99.5 (2106/2116) [99.1-99.7]**	100 (2116/2116) [99.8-100]	100 (2116/2116) [99.8-100]	99.9	99.8 (2137/2141) [99.5-99.9]	99.9 (2140/2141) [99.7-99.9]	100 (2141/2141) [99.8-100]
**Accuracy (%)**	99.9	99.4 (2164/2178)	99.8 (2174/2178)	99.9 (2177/2178)	99.9	99.8 (2173/2178)	99.9 (2176/2178)	100 (2178/2178)
**PPV (%)**	92.2	85.2 (58/68)	100 (58/58)	100 (61/61)	98.1	90.0 (36/40)	97.3 (36/37)	100 (37/37)
**NPV (%)**	99.9	99.8 (2106/2110)	99.8 (2116/2120)	99.9 (2116/2117)	99.9	99.9 (2137/2138)	99.9 (2140/2141)	100 (2178/2178)

Note. Data in parentheses were used to calculate the percentages, and data in brackets are 95 confidence intervals. * and † represents P-value <0.05 in McNemar test with reader 1 and reader 2, respectively. DLAp-r, deep learning-based algorithm per pixel-row; DLA, deep learning-based algorithm; PPV, positive predictive value; NPV, negative predictive value

Supplementary Table S2. External validation for spine CT anteroposterior topograms

	Spine implants				Hip implants			
	**DLAp-r**	**DLA**	**Reader 1**	**Reader 2**	**DLAp-r**	**DLA**	**Reader 1**	**Reader 2**
**Sensitivity (%)**	94.2	98.8 (241/244) [98.5-99.6]	98.4 (240/244) [95.9-99.4]	99.2 (242/244) [97.1-99.8]	88.6	94.1 (16/17) [73.0-98.9]	94.1 (16/17) [73.0-98-9]	100 (17/17) [81.6-100]
**Specificity (%)**	99.9	99.6 (270/271) [97.9-99.9]	99.6 (270/271) [97.9-99.9]	100 (270/271) [98.7-100]	99.7	*† **94.6 (471/498) [92.2-96.3]**	99.6 (496/498) [98.6-99.9]	100 (498/498) [99.2-100]
**Accuracy (%)**	99.4	99.2 (511/515)	99.0 (510/515)	99.6 (513/515)	99.7	94.6 (487/515)	99.4 (512/515)	100 (512/512)
**PPV (%)**	99.7	99.6 (241/242)	99.6 (240/241)	100 (242/242)	69.2	37.2 (16/43)	88.9 (16/18)	100 (17/17)
**NPV (%)**	99.4	98.9 (270/273)	98.5 (270/274)	99.3 (271/273)	99.9	99.8 (471/472)	99.8 (496/497)	100 (498/498)

Note. Data in parentheses were used to calculate the percentages. * and † represents P-value <0.05 in McNemar test with reader 1 and reader 2, respectively. AP, anteroposterior; DLAp-r, deep learning-based algorithm per pixel-row; DLA, deep learning-based algorithm; PPV, positive predictive value; NPV, negative predictive value

Supplementary Table S3. External validation for spine CT lateral topograms

	Spine implants				Hip implants			
	DLAp-r	DLA	Reader 1	Reader 2	DLAp-r	DLA	Reader 1	Reader 2
**Sensitivity (%)**	95.3	99.2 (242/244) [97.1-99.8]	98.4 (240/244) [95.7-99.4]	100 (244/244) [98.5-100]	NA	NA	100 (13/13) [77.2-100]	100 (13/13) [77.2-100]
**Specificity (%)**	99.9	99.6 (270/271) [97.9-99.9]	100 (271/271) [98.6-100]	100 (271/271) [98.6-100]	100	100 (502/502) [99.2-100]	100 (502/502) [99.2-100]	100 (502/502) [99.2-100]
**Accuracy (%)**	99.4	99.4 (240/243)	99.2 (511/515)	100 (515/515)	99.7	97.5 (502/515)	100 (515/515)	100 (515/515)
**PPV (%)**	98.6	99.6 (270/272)	100 (240/240)	100 (240/240)	NA	NA	100 (13/13)	100 (13/13)
**NPV (%)**	99.5	99.3 (242/243)	98.6 (271/275)	100 (275/275)	99.7	97.5 (502/515)	100 (502/502)	100 (502/502)

Note. Data in parentheses were used to calculate the percentages. * and † represents P-value <0.05 in McNemar test with reader 1 and reader 2, respectively. AP, anteroposterior; DLAp-r, deep learning-based algorithm per pixel-row; DLA, deep learning-based algorithm; PPV, positive predictive value; NPV, negative predictive value

Supplementary Figure S3. Usage of the algorithm results during reconstruction

The classification is calculated on the topogram to select the AiMAR preset for an optimized reconstruction of each slice

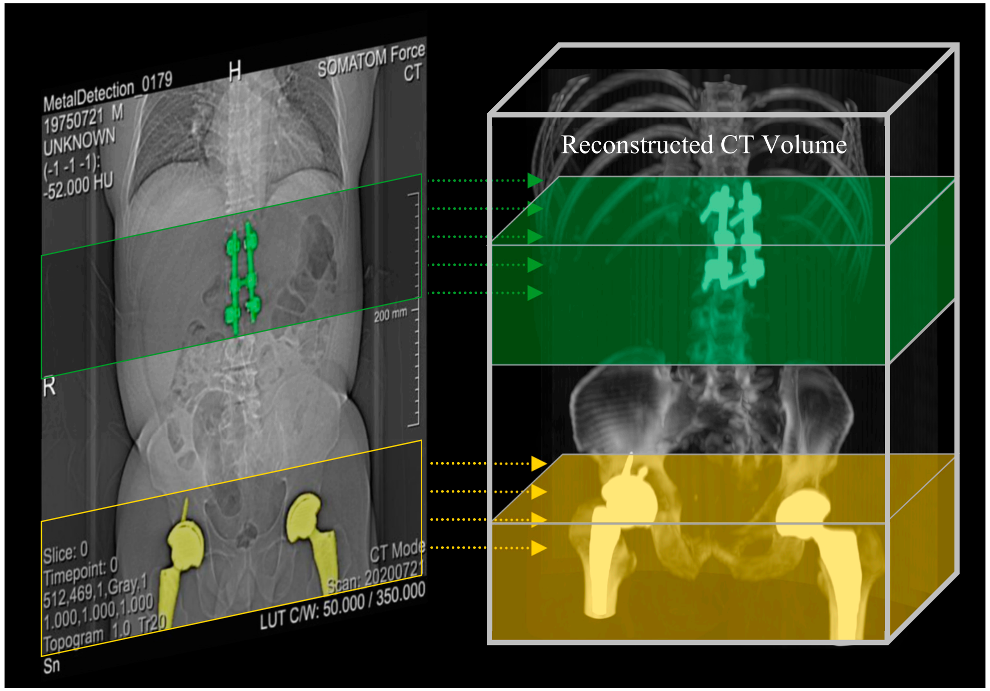

Supplement: Supplementary file 1 [file diagnostics-14-00668-s001.zip › diagnostics-2868906-supplementary.pdf]
